# Dissection of the genetic basis and molecular mechanism of ovule number per ovary in oilseed rape (*Brassica napus* L.)

**DOI:** 10.3389/fpls.2024.1489490

**Published:** 2025-01-28

**Authors:** Muslim Qadir, Xinyi Lin, Farhan Nabi, Kishore Kumar Ashok, Xue-Rong Zhou, Qingbin Sun, Peiman Shi, Xinfa Wang, Jiaqin Shi, Hanzhong Wang

**Affiliations:** ^1^ Oil Crops Research Institute of the Chinese Academy of Agricultural Sciences, Key Laboratory of Biology and Genetic Improvement of Oil Crops, Ministry of Agriculture and Rural Affairs, Wuhan, Hubei, China; ^2^ College of Agriculture, South China Agricultural University, Guangzhou, China; ^3^ Integrative Agriculture Department, College of Agriculture and Veterinary Medicine, United Arab Emirates University (UAEU), Abu Dhabi, United Arab Emirates; ^4^ Commonwealth Scientific and Industrial Research Organization (CSIRO) Agriculture Food, Canberra, ACT, Australia

**Keywords:** ovule number per ovary, QTL mapping, differentially expressed genes, phytohormones, candidate genes, *Brassica napus*

## Abstract

Ovule number per ovary (ONPO) determines the maximum potential of seed number per fruit that is a direct component of seed yield in crops. This study aimed to dissect the genetic basis and molecular mechanism of ONPO using a newly developed doubled haploid (DH) population in oilseed rape. In all the four investigated environments, the ONPO of 201 DH lines exhibited normal distribution with a wide variation from 22.6 to 41.8, suggesting quantitative inheritance appropriate for mapping QTL. A skeleton genetic map of 2111 markers within 19 linkage groups was developed, with a total of 1715.71 cM in length and an average of 0.82 cM between markers. Linkage mapping identified ten QTLs that were distributed on eight chromosomes and explained 7.0-15.9% of the phenotypic variance. Among these, four were identical to the reported and two were repeatedly detected with relatively large effects, highlighting their potential for marker-assisted selection. Phytohormone quantification of ovaries (at the ovule initiation stage) from two pools of high and low ONPO lines showed significant differences in the levels of nine sub-types of phytohormones, suggesting their important roles in regulating ovule number. Transcriptomic analysis identified 7689 differentially expressed genes (DEGs) between the two pools, of which nearly half were enriched into functional categories of reported genes regulating ONPO, including protein, RNA, signalling, miscellaneous, development, hormone metabolism, and tetrapyrrole synthesis. Integration of linkage QTL mapping, transcriptome sequencing and BLAST analysis identified 15 homologues of reported ovule number genes and 327 DEGs in the QTL regions, which were considered as direct and potential candidate genes. These findings propose further insights into the genetic basis and molecular mechanisms of ONPO, which will facilitate future gene cloning and genetic improvement for enhancing seed yield in oilseed rape.

## 1 Introduction

Oilseed rape (*Brassica napus* L.) is widely cultivated worldwide as an important oil crop after soybean, which provides edible oil to humans and animals and biofuel for industrial production (Chalhoub et al., 2014; Shi et al., 2015; Yang et al., 2017; Qadir et al., 2022). With global population increase, there is urgent need to improve the production of oilseed rape to meet the growing demand for edible oil and biofuel (Ahmad et al., 2023; Hu et al., 2017). However, the global oilseed rape yield per unit (≈2 ton/ha) has stagnated in the past decade (https://apps.fas.usda.gov/psdonline). Breaking the bottleneck of oilseed rape yield relies on the full understanding of it as well as the exploration and utilization of the high-yielding genes.

The seed yield per plant in oilseed rape is equal to the multiplication of its three components, i.e. number of siliques per plant, number of seeds per silique, and seed weight. The number of seeds per silique is multiplicatively determined by the number of ovules per ovary, the fertilization rate of ovules and the rate of fertilized ovules developed into seeds (Yang et al., 2017; Qadir et al., 2022). In flowering plants, ovules are the precursors of seeds, which contain the female gametophytes and develop into seeds after fertilization (Drews and Koltunow, 2011; Yuan and Kessler, 2019). The ovule number per ovary (ONPO) varies greatly across the different species and even among the different varieties of same species (Burd et al., 2009; Khan et al., 2019; Qadir et al., 2022). Different with seed number per silique, the ONPO is unable to be simply observed by the naked eye and not to be directly selected by breeders. As a result, the ONPO of oilseed rape cultivars is much less than the its max in the germplasm resources (Qadir et al., 2022), which probably represents an unselected character that can potentially enhance the seed yield of oilseed rape. Thus, increasing ONPO could be an effective strategy to enhance seed yield for ensuring food security (Jiao et al., 2021), which largely depends on the understanding of its genetic mechanism (Yuan and Kessler, 2019; Qadir et al., 2022).

The previous studies have showed that the size and position of flowers (Gomez et al., 2018; Yuan and Kessler, 2019), the availability of nutrients (Strelin and Aizen, 2018), and the levels of phytohormones may affect ONPO (Barro-Trastoy et al., 2020; Qadir et al., 2022). In *Arabidopsis* and other species, more than 70 genes have been reported to regulate ONPO (Qadir et al., 2021), which are involved in multiple processes of ovule development including the formation of carpel meristem, the identity of ovule, the initiation of primordia, and the development of integuments (Skinner et al., 2004; Qiao and Yang, 2011; Qadir et al., 2022; Ahmad et al., 2023). The reported genes regulating ONPO form an integrated molecular network, in which four types of phytohormones play a core role. Of them, AUX, BR and CK positively regulate ONPO, whereas GA has negative effect (Nemhauser et al., 2000; Yuan and Kessler, 2019; Qadir et al., 2022).

By linkage and/or association QTL mapping, many studies have identified nearly two hundred QTLs for seed number per silique in oilseed rape (Zhu et al., 2020). In contrast, only a few studies have reported about thirty QTLs for ONPO (Khan et al., 2019; Qadir et al., 2022; Ahmad et al., 2023), therefore the genetic basis of ONPO remains largely unknown. Transcriptomic analysis can detect the gene expression abundance at given stage and/or tissue and identify the differentially expressed genes, which may be the upstream or downstream genes in regulating a specific development process. Lot of research have demonstrated that the integration of QTL mapping with transcriptomic analysis is an effective strategy for screening candidate genes (Wang et al., 2020; Qadir et al., 2022; Qadir et al., 2022).

The current study aimed to dissect the genetic basis and molecular mechanism of ONPO by using a newly developed DH population in *B. napus*. The DH population was planted in multiple environments to identify QTL for ONPO. In addition, two pools of high and low ONPP lines were subjected to the comparative analysis of phytohormones and transcriptome at the initial stage of ovule development. More importantly, the candidate genes underlying ONPO QTL were identified by integrating the results of linkage QTL mapping and comparative transcriptomic analysis. These data provide useful information for the further gene cloning and molecular improvement of ONPO in oilseed rape.

## 2 Materials and methods

### 2.1 Population construction and field experiment

The DH population consisting of 201 lines was originated from two Spring and semi-Winter rapeseed varieties 3S1181 and 3S1235, which were chosen from an association population constructed in our lab (Qadir et al., 2022). Field experiments were conducted in the typical spring and semi-Winter rapeseed planting areas across three years, including Ping’an in Qinghai province (32.27° N, 114.52° E) in 2020 (coded as 20QH), Yangluo in Wuhan city (29.58° N, 113.53° E) in 2021 and 2022 (coded as 21YL and 22YL), and Huanggang city in Hubei province (30.44° N, 114.87° E) in 2022 (coded as 22HG). The field experiments were conducted according to the procedure described previously (Qadir et al., 2022).

### 2.2 Phenotypic investigation of ovule number per ovary

For investigating ONPO, three buds on the main inflorescence of five plants were randomly sampled at the beginning of flowering, which resulted in 15 buds for each replication. The formalin solution was used to fix and store the samples buds (Yang et al., 2017). Petals and stamens were removed by tweezers and the ovary was taken out, according to the previously published experimental procedures (Qadir et al., 2022).

### 2.3 Quantification of phytohormones

In order to examine whether the variation of ONPO is related to the levels of endogenous phytohormones, auxin (AUX), Brassinolide (BR), cytokinin (CK) and gibberellin (GA) in the ovaries (at the initial stage of ovule development) of two pools of high and low ONPO lines were measured. The buds between 0.5 to 1 mm length were selected for dissection by tweezers under microscope. The frozen ovaries from high or low ONPO lines in each biological repeat were equally mixed to produce the corresponding pools. The abundance of phytohormones was detected with phytohormonal platform at Metware Biotechnology Company (Wuhan, China).

### 2.4 Genetic linkage map construction

The two parents and 201 DH lines were genotyped using the 50K SNP array comprising of 45,707 SNP markers, which resulted in a total of 11348 polymorphic markers. Moreover, after removing the markers with heterozygosity >5% or missing rate >20%, a total of 7176 high-quality SNP markers were obtained for subsequent analysis. The genetic linkage analysis was performed using JoinMap 4.0 software (Van Ooijen, 2006) with the default parameters: goodness of fit ≤5.0, LOD scores >2.0, recombination frequency < 0.4.

### 2.5 Linkage mapping of QTL

Linkage mapping of QTL was performed using CIM (composite interval mapping) method in WinQTLCart2.5 software with the default setting (Zeng, 1994). The LOD (logarithm of the odds) threshold for defining a significant QTL was determined by permutation test (Churchill and Doerge, 1994) with 1000 repetitions at P < 0.05. The LOD value, additive effect (A), confidence interval (CI), and phenotypic variance explained (PVE) of each QTL were estimated.

### 2.6 RNA sequencing and transcriptomic analysis

The dissected ovaries (at the initial stage of ovule development) from high or low ONPO lines were equally mixed to produce the corresponding pools with three biological repeats (M1-M3 and L1-L3). The total RNA of each sample was isolated using the RNeasy Plant Mini Kit (Qiagen Inc., Germany). The cDNA library was constructed and sequenced using DNBSEQ-T7 platform of BGI Genomics Co., Ltd (Shenzhen, China).

The low-quality raw reads were removed by filtering with SOAPnuke v1.4.0 software (Chen et al., 2018), and the remaining clean reads were used for subsequent analysis. The clean reads were mapped to Darmor-bzh reference genome using HISAT v2.1.0 software (Kim et al., 2015). The transcripts of each sample were re-constructed and integrated by StringTie and Cuffmerge software respectively (Shumate et al., 2022). The gene expression abundance in each sample was estimated using RSEM v1.12.8 software (Li and Dewey, 2011). Both the P-value 0.005 and Q-value 0.05 were simultaneously used to define the deferentially expressed genes (DEGs). The clean reads of the six samples were uploaded to NCBI database with accession number PRJNA1150986.

The classification and enrichment analysis of Gene Ontology (GO) terms and Kyoto Encyclopedia of Genes and Genomes (KEGG) pathways were performed using the online bioinformatics analysis platform of BGI Genomics Co., Ltd (https://biosys.bgi.com). The functional classification of DEGs were conducted by online tool Super-Viewer (http://bar.utoronto.ca/ntools/cgi-bin/ntools_classification_superviewer.cgi).

### 2.7 Validation of DEGs through qRT-PCR

To validate the reliability of DEGs identified by RNA sequencing, qRT-PCR was performed using the same RNA for sequencing. The M-MLV reverse transcriptase (Promega) was used to synthesize cDNA containing total RNA (4μg) and oligo (dT) primers, as specified by the company instructions. The qRT-PCR experiment was performed using SYBR Green Real-time PCR Master Mix (Toyobo, Osaka, Japan) and a Bio-Rad CFX96 Real-Time Detection System. The relative expression was calculated using the 2−ΔΔCT method, with *B. napus* ACTIN2 as an internal control (Qadir et al., 2022).

### 2.8 Data analysis

The phenotypic data of the ONPO was statistically analyzed using Microsoft Excel, including mean, standard deviation, and coefficients of variation etc. The phenotypic correlation and variance were respectively analyzed using the CORR and ANOVA procedure in SAS software. The broad-sense heritability was estimated using the following formula: *h* ^2^ = σ_g_^2^/(σ_g_^2^ + σ_ge_^2/^n + σ_e_^2^/nr), where σ_g_^2^, σ_ge_^2^ and σ_e_^2^ respectively represented the variances caused by genotype, genotype×environment and error, while n and r respectively represented the environment and replicate number.

## 3 Results

### 3.1 Phenotypic variation of DH population and parents

**Figure 1:**
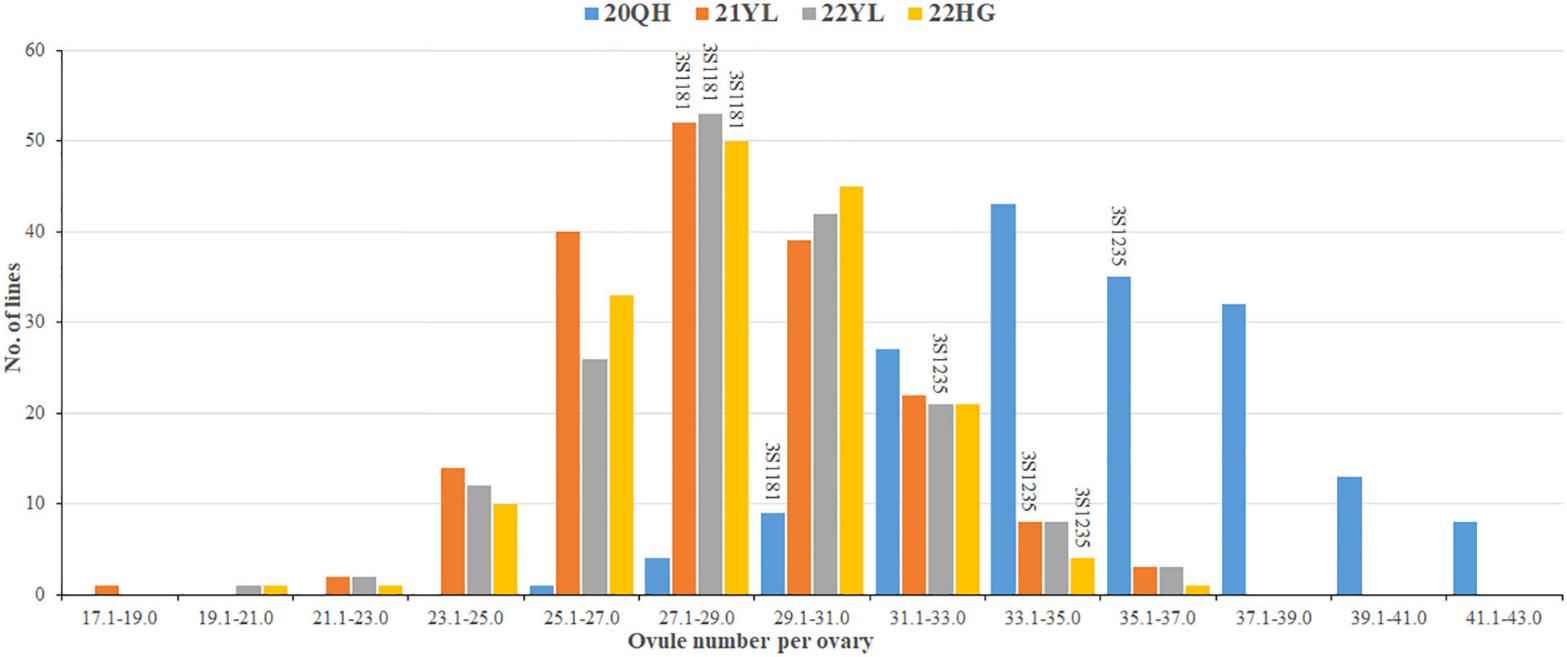
Frequency distribution of over number per ovary of the DH population and both parents planted in four environments. The horizontal axis shows the number of ovules per ovary that are evenly divided into a dozens of groups. The vertical axis shows the number of lines in DH population. The height of columns on the figure show the number of DH lines, and the text on the columns represent the codes of two parents. The different colors of columns distinguished four investigated environments as shown in the legends. The details of four environments are described in the first section of Materials and Methods.

The two parents and 201 DH lines were planted in four environments including 20QH, 21YL, 22YL and 22HG. Of these, 20QH belongs to Spring type environment and seeds were sown at the middle of May whereas 21YL, 22YL and 22HG belong to semi-Winter type environment and seeds were sown at the end of September. The ONPO of parent 3S1235 in four investigated environments ranged from 32.7 to 36.8 in average, and was significantly more than that of another parent 3S1181 from 27.2 to 29.3 in average (**Table 1**). In each of the four investigated environments, the ONPO range of DH population exceeded that of two parents, indicating that favorable alleles should be present in both parents. Interestingly, the ONPO mean (35.3) of DH population in 20QH was much higher than those in other three environments, suggesting that oilseed rape tended to differentiate more ovules in Spring than semi-Winter type growing areas. As expected, the ONPO of DH population showed normal distribution in all four investigated environments (**Figure 1**), exhibiting quantitative genetic characteristics.

**Table 1:** The descriptive statistics of ONPO for DH population and its two parents in four investigated environments.

Environments^*^	Parents			DH population				
	3S1181	3S1235	P_t-test_	Mean± SD	Min	Max	CV (%)	Skewness
20QH	29.3 ± 0.72	36.8 ± 0.67	2.63E-03	35.3 ± 3.2	26.4	42.3	9.1	0.03
21YL	28.0 ± 0.85	35.0 ± 0.57	8.05E-03	28.9 ± 3.1	17.8	36.9	10.8	0.62
22YL	27.2 ± 0.65	32.7 ± 0.57	2.12E-04	28.9 ± 2.6	19.3	36.5	8.8	0.49
22HG	27.4 ± 0.46	34.5 ± 0.61	7.32E-05	28.6 ± 2.2	20.0	36.8	7.6	0.10

*For the details of environments, please see the first section of Materials and Methods.

Generally, the ONPO of DH population in three semi-Winter rapeseed environments (21YL, 22YL and 22HG) were highly correlated (r=0.87, 0.85 and 0.85), but they exhibited low correlation with Spring rapeseed environment 20QH (r=0.39, 0.28 and 0.38) (**Supplemenmtary Table S1**), which suggested that ONPO might vary across the different environments. The results of variance analysis demonstrated that genotype (G), environment (E), and their interaction (G × E) were all significant on ONPO (**Supplementary Table S2**). The calculated broad-sense heritability for ONPO was as middle as 0.52, likely due to the large environmental difference in 20QH.

### 3.2 Construction of genetic linkage map

**Figure 2:**
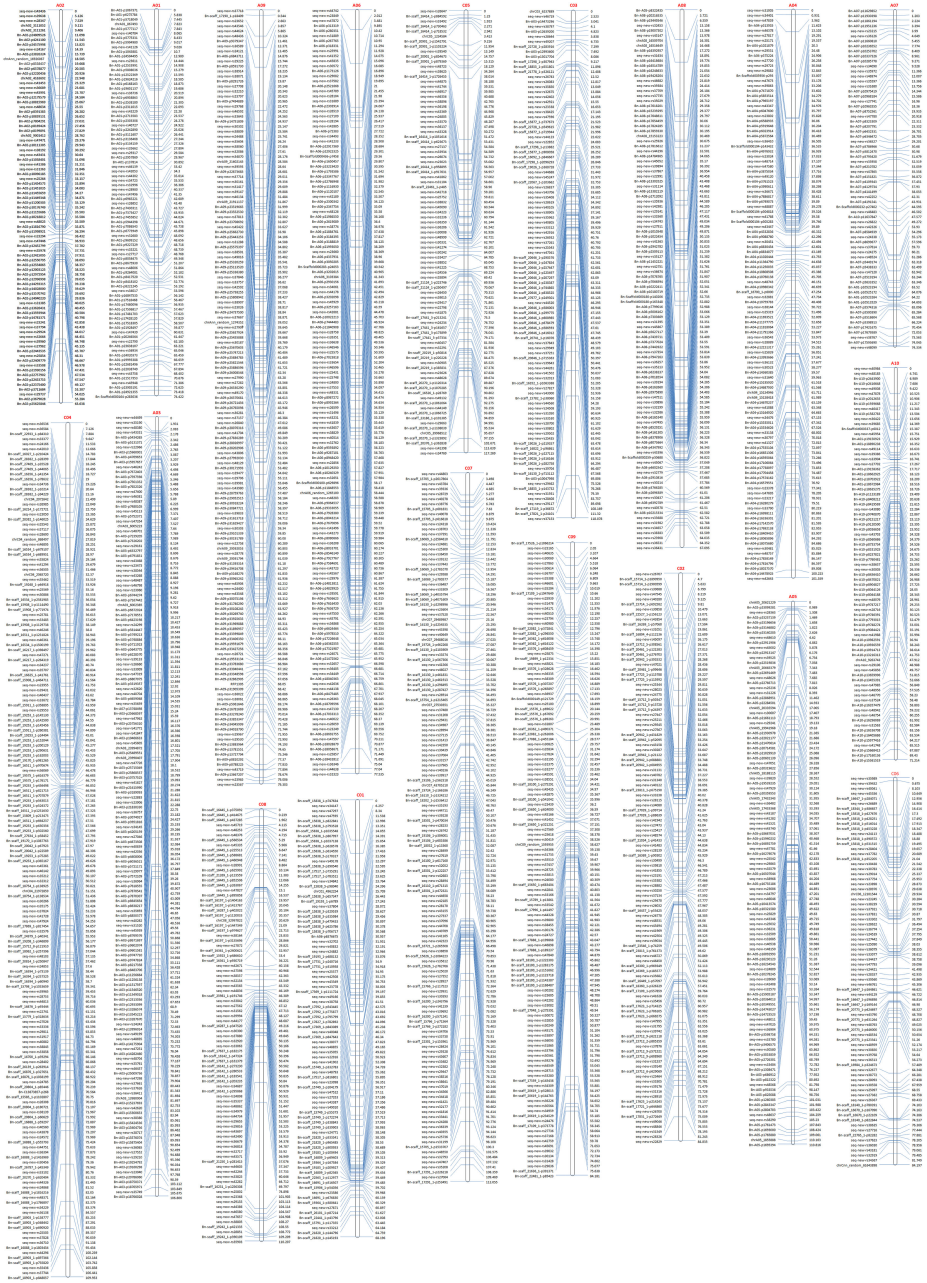
The skeleton genetic linkage map of 19 linkage groups with 2111 markers. The names of 19 assigned pseudo-chromosomes (A01-A10; C01-C09) are shown on the top of each linkage group. The names and genetic distance of the SNP markers are respectively drawn on the left and right side of each linkage group.

The remaining high-quality SNP markers were subjected to linkage grouping and resulted in a skeleton genetic map of 2111 SNP markers within 19 linkage groups (**Figure 2**; **Table 2**). The number of SNP markers in 19 linkage groups varied from 68 (A07) to 171 (C04), with an average of 111. The genetic length of 19 linkage groups varied from 63.6 (A02) to 118.1 (C3) cM, with a total length of 1715.7 cM. The marker density of 19 linkage groups varied from 0.76 (C05) to 1.95 (A09), with a mean of 1.23 per cM. The physical length of 19 linkage groups varied from 15.8 (A10) to 54.4 (C3) Mb, with a total length of 587.9 Mb. The covered ratios of 19 linkage groups varied from 63.6% (C06) to 100.0% (C04), with a mean of 91.5%. It was obvious that the recombination frequencies of the 10 linkage groups in A sub-genome (2.63 to 5.36, mean=3.94) were mostly higher than the 9 linkage groups in C sub-genome (1.79 to 3.58, mean=2.35), with a mean of 2.92 per Mb for the whole genetic map.

**Table 2:** The summary statistics of the constructed genetic linkage map of DH population.

Linkage Group	Number of SNP	Genetic Distance	Marker density ^a^	Assembled length	Physical Distance	Coverage ratio ^b^	Recombination Frequency ^c^
A01	85	74.4	1.14	22.8	19.7	86.2%	3.79
A02	83	63.6	1.30	24.8	21.8	87.8%	2.92
A03	159	106.8	1.49	29.5	21.6	73.2%	4.95
A04	114	101.6	1.12	19.1	18.9	99.2%	5.36
A05	109	110.8	0.98	22.8	21.0	92.1%	5.28
A06	153	77.5	1.97	24.4	24.3	99.5%	3.19
A07	68	74.3	0.91	23.9	18.8	78.5%	3.96
A08	108	67.1	1.61	18.8	17.7	94.0%	3.79
A09	155	79.3	1.95	33.7	30.2	89.6%	2.63
A10	79	71.2	1.11	17.2	15.8	92.1%	4.50
A subgenome	1113	826.7	1.35	237.0	209.7	88.5%	3.94
C01	88	68.2	1.29	38.4	36.3	94.5%	1.88
C02	114	84.8	1.34	46.2	45.6	98.7%	1.86
C03	102	118.1	0.86	60.5	54.4	89.9%	2.17
C04	171	110.0	1.56	48.9	48.9	100.0%	2.25
C05	89	117.3	0.76	43.1	41.9	97.3%	2.80
C06	78	84.2	0.93	37.0	23.5	63.6%	3.58
C07	142	112.1	1.27	44.6	44.2	99.1%	2.53
C08	87	110.2	0.79	38.5	36.3	94.4%	3.03
C09	127	84.2	1.51	48.3	47.0	97.3%	1.79
C subgenome	998	889.0	1.12	405.5	378.2	93.3%	2.35
**Total**	**2111**	**1715.7**	**1.23**	**642.5**	**587.9**	**91.5%**	**2.92**

a Marker density is calculated as number of Bin/SNP markers divided by physical distance (Mb).

b Coverage ratio is calculated as the covered physical distance (Mb) of linkage groups divided by the length (Mb) of the assembled pseudo chromosomes.

c Recombination frequency is calculated as the genetic distance (cM) divided by physical distance (Mb).LOD Value stands for Logarithm of the Odds.

### 3.3 Linkage QTL mapping for ovule number per ovary

**Figure 3:**
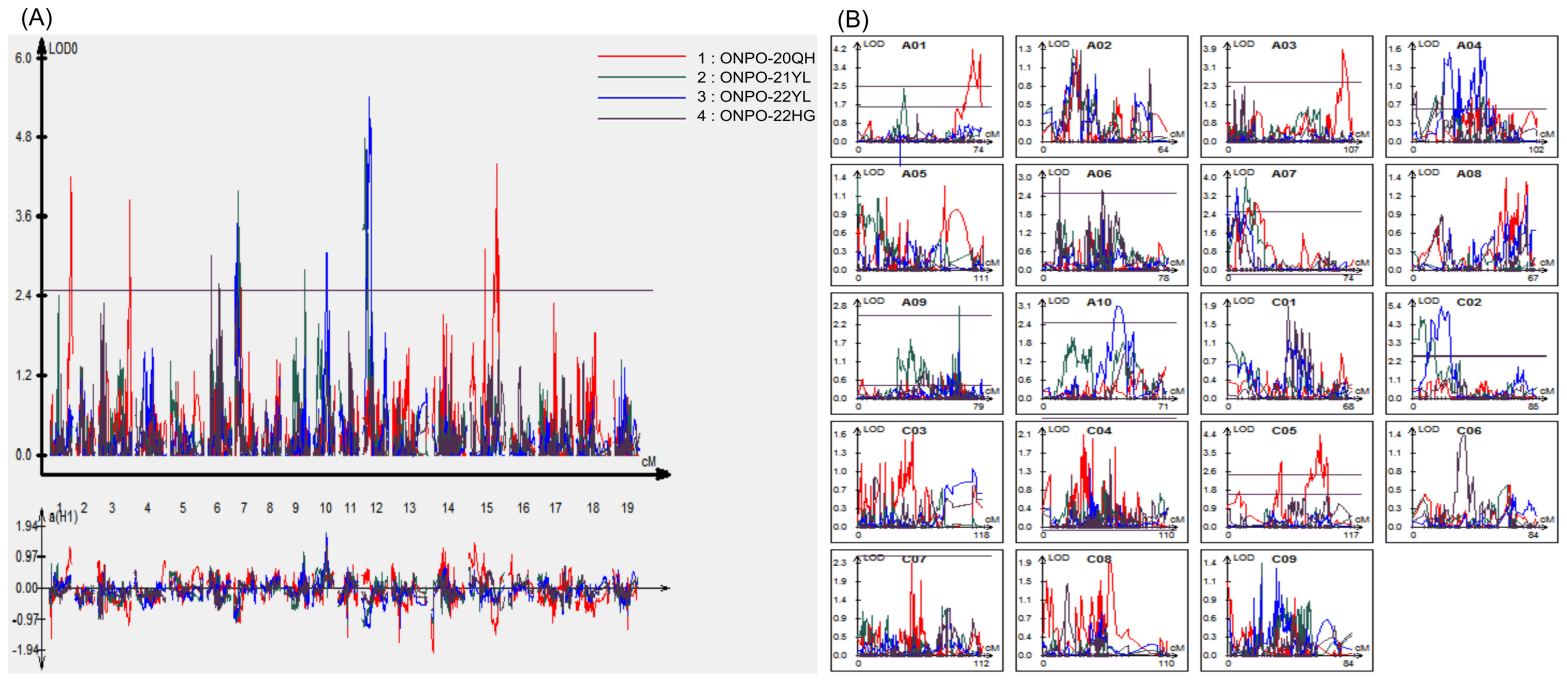
QTL scanning curves of ovule number per ovary for the DH population across four investigated environments. **(A)** The QTL LOD profiles of all 19 linkage groups across four environments. The horizontal and vertical axes show the 19 linkage groups and LOD scores, respectively. The LOD curves of four environments were distinguished by the different colors as shown in the legends. **(B)** The QTL LOD profiles for each of the 19 linkage groups, respectively. The horizontal and vertical axes show the genetic distance and LOD scores, respectively.

A total of 10 consensus QTLs of ONPO were identified in the DH population across four investigated environments (**Figure 3**; **Table 3**). Of these, 5, 3, 3 and 2 QTLs were identified from 20QH, 21YL, 22YL and 22HG environments, respectively. These QTLs were distributed in A01, A03, A06, A07, A09, A10, C02, and C05 linkage groups, accounting for 7.0-15.9% of the phenotypic variance. The additive effects of these QTLs could be either positive or negative, ranging from -1.16 to 1.32, which was highly accordant with the over-parent distribution of ONPO in DH population.

**Table 3:** The consensus QTL of ONPO detected in the DH population across four environments.

QTL name	LOD Value	R^2^ (%)	Genetic Position	Confidence Interval	Additive effect	Environment	Comparative QTL analysis
*qONPO.A01-1*	4.23	11.3	67.91	66.7-70.7	1.32	20QH	Qadir et al., 2022
*qONPO.A03-1*	3.88	11	97.81	96.8-102.5	1.11	20QH	Novel
*qONPO.A06-1*	3.02	8.9	11.31	11.2-11.7	0.68	22HG	Novel
*qONPO.A06-2*	2.61	8	37.01	36.5-37.5	0.64	22HG	Novel
*qONPO.A07-1*	3.52	10.9	5.51	0-7.5 2.5-13	-1.00	22YL	Novel
	4.01	11.6	11.41		-1.00	21YL	
	2.93	8.2	17.01	15.6-19.9	0.99	20QH	
*qONPO.A09-1*	2.81	7	63.91	63.7-64.6	1.17	21YL	Novel
*qONPO.A10-1*	3.06	13.2	42.81	38.3-47.4	1.06	22YL	Khan et al., 2019; Ahmad et al., 2023
*qONPO.C02-1*	4.8	14.9	5.61	0-6.8 12.2-17.5	-1.16	21YL	Khan et al., 2019; Qadir et al., 2022
	4.62	15.9	13.11		-1.09	22YL	
*qONPO.C05-1*	3.13	8.4	50.31	47.9-51.2	1.1	20QH	Novel
*qONPO.C05-2*	4.42	14.1	86.51	85.5-89.1	1.4	20QH	Khan et al., 2019

It should be noted that two QTLs in A07 and C02 linkage groups (*qONPO.A07-1* and *qONPO.C02-1*) were repeatedly detected in different environments and explained a largely 11.3% and 15.4% of the phenotypic variance (**Table 3**), which should be considered as major QTLs. About thirty QTLs of ONPO have been previously reported by linkage or association mapping in oilseed rape (Khan et al., 2019; Qadir et al., 2022; Ahmad et al., 2023). The results of comparative QTL analysis (**Supplementary Table S3**) showed that four QTLs (*qONPO.A01-1*, *qONPO.A10-1*, *qONPO.C02-1*, *qONPO.C05-2*) were overlapped with the reported ONPO QTLs, while the remaining six should be novel loci. For example, the genomic region *qONPO.A01-1* detected in the present study was overlapped with two association loci identified in our previous study (Qadir et al., 2022).

### 3.4 Comparison of phytohormones level between high and low ONPO lines

The previous studies have showed that four phytohormones (AUX, BR, CK, and GA) play a central role in regulating ONPO (Qadir et al., 2021). Therefore, the concentrations of these four phytohormones in ovaries at ovule initiation stage were measured and compared between high and low ONPO pools (**Table 4**). The two pools respectively comprised ten high and nine low ONPO lines that were chosen from top or bottom 10% lines common in the investigated Spring and semi-Winter rapeseed environments, with an average difference of 12.6 and 9.4, respectively.

**Table 4:** Comparison of endogenous phytohormones contents between two pools of high and low ONPO (H *vs* L) lines.

Category	Subclass	H pool	L pool	P_t.test_
**AUX**	IAM	3.52 ± 0.61	5.19 ± 1.06	0.04
	IAA-Leu-Me	0.26 ± 0.15	0.27 ± 0.08	0.49
	IAA-Leu	0.49 ± 0.08	0.72 ± 0.22	0.08
	IAA-Glu-diMe	1.45 ± 1.23	1.62 ± 0.60	0.42
	IAA-Glu	0.59 ± 0.15	0.60 ± 0.13	0.47
	IBA	1.46 ± 1.20	1.47 ± 0.50	0.5
	MEIAA	16.29 ± 3.54	16.41 ± 2.46	0.48
	IPA	9.83 ± 5.84	8.22 ± 2.40	0.34
	ILA	4.90 ± 0.02	5.35 ± 0.30	0.03
	IAA-Val	0.64 ± 0.07	1.06 ± 0.54	0.12
	IAA-Gly	0.92 ± 0.02	1.18 ± 0.30	0.1
	IAA-Asp	8.07 ± 0.42	6.75 ± 1.63	0.12
	IAA	16.42 ± 0.95	16.9 ± 4.12	0.43
**BR**	28-homoCS	0.03 ± 0.01	0.04 ± 0.01	0.38
	28-norBL	0.08 ± 0.01	0.06 ± 0.00	0.01
	28-norCS	0.54 ± 0.07	0.41 ± 0.12	0.1
	6-deoxoCS	14.96 ± 2.59	13.7 ± 0.17	0.23
	BL	1.68 ± 0.61	0.91 ± 0.32	0.06
	CS	2.63 ± 0.34	2.41 ± 0.43	0.26
	TY	0.20 ± 0.02	0.18 ± 0.01	0.04
**CK**	2MeScZR	0.60 ± 0.14	0.79 ± 0.14	0.09
	K9G	0.49 ± 0.24	0.45 ± 0.17	0.41
	DHZ7G	0.06 ± 0.01	0.08 ± 0.01	0.03
	IP	0.04 ± 0.02	0.03 ± 0.01	0.29
	IPR	4.80 ± 0.30	6.25 ± 1.22	0.06
	K	0.11 ± 0.04	0.08 ± 0.03	0.18
	cZ	0.15 ± 0.05	0.14 ± 0.06	0.39
	cZR	11.41 ± 3.07	13.1 ± 1.74	0.22
	cZROG	0.96 ± 0.04	0.97 ± 0.02	0.37
	iP7G	1.48 ± 0.23	1.70 ± 0.31	0.19
	tZOG	1.63 ± 0.08	1.47 ± 0.13	0.07
	DZ	0.24 ± 0.05	0.21 ± 0.02	0.18
	mT9G	0.23 ± 0.08	0.17 ± 0.05	0.18
	tZR	0.76 ± 0.07	0.47 ± 0.13	0.01
	iP9G	0.41 ± 0.09	0.60 ± 0.15	0.07
**GA**	GA1	0.43 ± 0.01	0.33 ± 0.00	0
	GA3	0.23 ± 0.02	0.31 ± 0.17	0.28
	GA4	2.30 ± 0.45	1.77 ± 0.61	0.15
	GA5	0.13 ± 0.04	0.17 ± 0.01	0.15
	GA6	3.63 ± 0.43	3.59 ± 1.66	0.49
	GA8	1.34 ± 0.36	1.10 ± 0.56	0.29
	GA9	0.57 ± 0.12	0.34 ± 0.10	0.03
	GA15	0.43 ± 0.55	0.28 ± 0.44	0.37
	GA20	0.36 ± 0.09	0.34 ± 0.08	0.42
	GA24	1.59 ± 0.34	0.89 ± 0.11	0.01
	GA29	1.04 ± 0.28	1.65 ± 0.70	0.12
	GA34	5.54 ± 1.10	5.60 ± 1.18	0.48
	GA51	0.43 ± 0.17	0.28 ± 0.08	0.11

AUX, Auxins; BR, Brassinosteroid; CK, Cytokinins; GA, Gibberellins; H Pool, High Pool; L Pool, Low Pool.

For auxin, the concentrations of IAM and ILA in H pool (3.52 and 4.90 pg/mg) were significantly lower than those in L pool (5.19 and 5.35 pg/mg). For brassinosteroid, the levels of 28-norBL and TY in H pool (0.08 and 0.20 pg/mg) were significantly higher compared to L pool (0.06 and 0.18 pg/mg). For cytokinin, the contents of DHZ7G and tZR in H pool (0.06 and 0.76 pg/mg) were respectively lower and higher than that in L pool (0.08 and 0.47 pg/mg). For gibberellin, the levels of GA1, GA9 and GA24 in H pool (0.43, 0.57 and 1.59 pg/mg) were significantly higher than those in L pool (0.33, 0.34 and 0.89 pg/mg). In conclusion, the concentrations of nine sub-types of AUX, BR, CK, and GA showed significant difference between H and L pools, which should be responsible for the ONPO difference in these lines.

### 3.5 Comparative transcriptomic analysis between high and low ONPO lines

#### 3.5.1 Overview of the transcriptome of two pools of high or low ONPO lines

To further reveal the molecular mechanisms underlying the natural variation of ovule number in the DH population, two pools of high and low ONPO lines were subjected to a comparative transcriptomic analysis using the ovaries at the initial stage of ovule development. **Supplementary Table S4** showed a summary of the transcriptome data of six samples (two pools and each with three biological repeats). After filtrating out the raw reads with low-quality, a total of 259,097,198 clean reads with 38.87 Gb clean data were obtained from these six samples (codes: M1 to M3 and L1 to L3). The total mapping-genome ratio of six samples ranged from 82.09% to 85.07%, with a mean of 84.13%. The Q30 and GC content of these six samples varied from 94.22 to 94.84% and from 56.43 to 64.32%, with a mean of 94.53% and 61.56% respectively.

**Figure 4:**
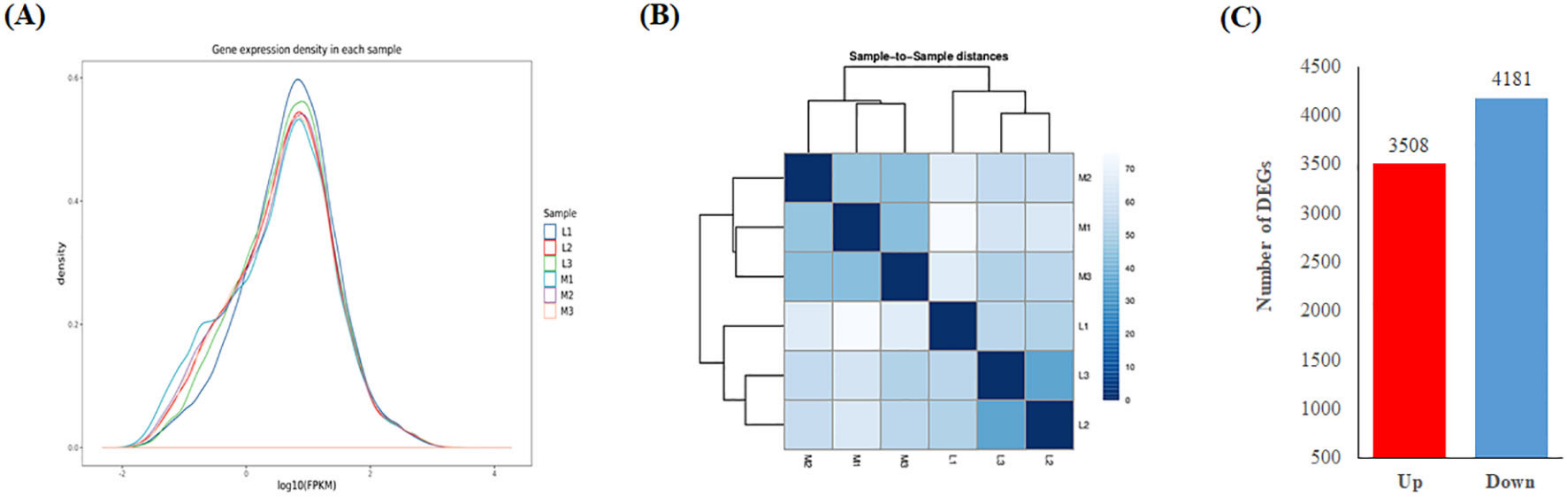
Statistics of gene expression in two pools of high and low ONPO lines. **(A)** The gene expression density in six samples. The horizontal and vertical axes show the log10(FPKM) and density respectively. **(B)** The correlation heat map of gene expression among the six samples (M1-3 *vs*. L1-3). The correlation coefficients are displayed in color column as shown in legends. **(C)** The statistic of DEGs number between the two pools. The horizontal and vertical axes show the up/down pattern and number of DEGs, respectively.

The distribution of Log_10_FPKM value of the expressed genes in six samples were similar (**Figure 4A**), which was highly accordant with the fact that these samples were from the same tissue. Obviously, the correlation of transcriptome between the different duplications of the same pool was higher than that between different pools (**Figure 4B**). A total of 78484 expressed genes were detected in the two pools, of which 7689 genes were differentially expressed between two pools (**Figure 4C**; **Supplementary Table S5**). Of these differentially expressed genes (DEGs), 3508 and 4181 were respectively up- and down-regulated, in M compared to L pool. In order to validate the reliability of these DEGs, qRT-PCR were performed using specific primers for ten randomly selected DEGs and three most likely candidate genes (**Supplementary Table S6**). The result showed that all of these DEG exhibited comparable patterns of expression with the RNA-seq data excluding *BnaC05g21200D*, although the fold of change varied between the two methods (**Supplementary Figure S1**).

#### 3.5.2 Go classification and KEGG enrichment analysis of DEGs

**Figure 5:**
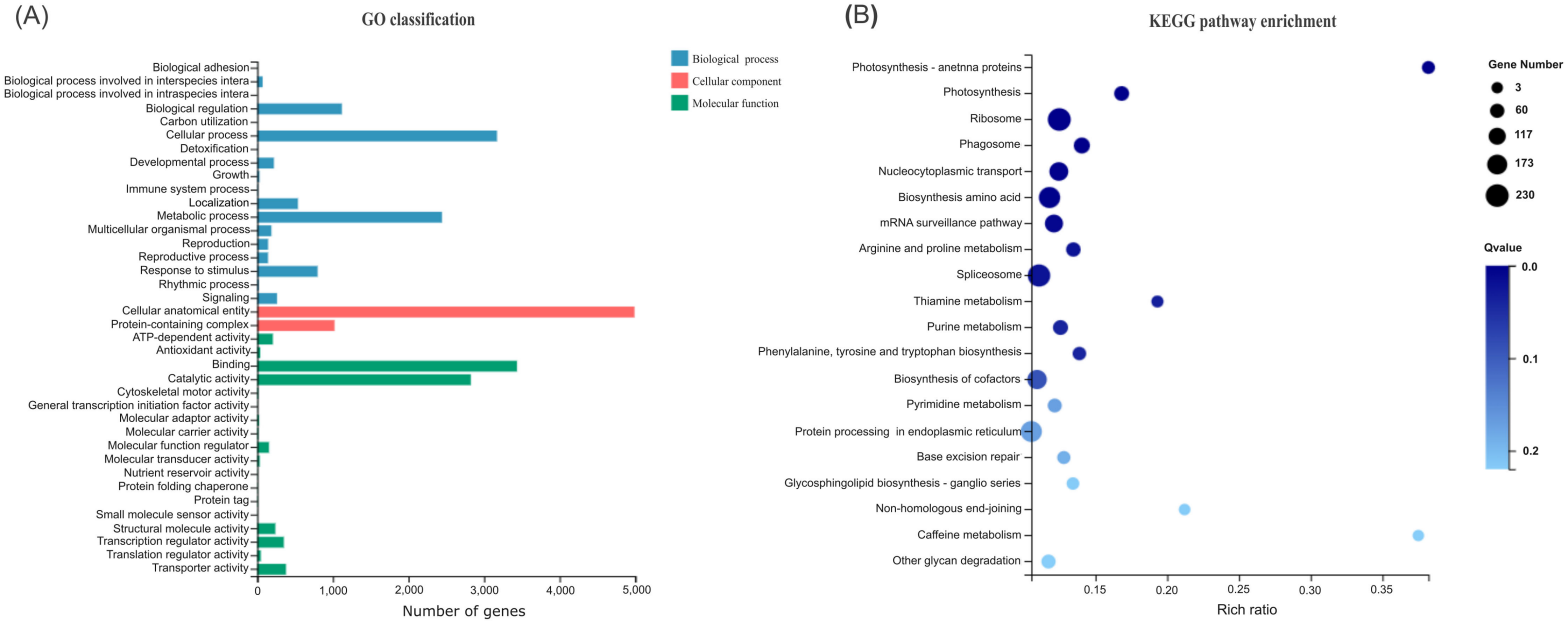
GO classification **(A)** and KEGG **(B)** pathway enrichment of DEGs. The vertical axis shows the GO categories and KEGG pathways, respectively. The horizontal axis represents the gene number and rich ratio, respectively.

All 7689 DEGs were first subjected to gene ontology (GO) classification including biological process, cellular component and molecular function (**Figure 5A**). The biological functions of DEGs were classified into 18 categories, mainly including biological regulation, cellular process, localization, metabolic process, and response to stimulus. The cellular components of DEGs were classified into two categories, i.e. cellular anatomical entity and protein-containing complex. The molecular functions of DEGs were classified into 18 categories, mainly comprising ATP-dependent activity, binding, catalytic activity, molecular function regulator, structural molecular activity, transcription regulator activity, and transporter activity.

Then these DEGs were subjected to Kyoto Encyclopedia of Genes and Genomes (KEGG) pathway enrichment analysis. They were significantly enriched into 12 pathways at Q-value < 0.05 (**Figure 5B**). Notably, about half of the enriched pathways (arginine and proline metabolism, biosynthesis of amino acids, phenylalanine, tyrosine and tryptophan biosynthesis, mRNA surveillance, nucleocytoplasmic transport, ribosome, spliceosome) are related to protein, providing material foundation for ovule development. Interestingly, two enriched pathways are related to photosynthesis, supplying the energy for ovule development. The purine metabolism is related to nucleic acid and energy, both of which are required by ovule development.

#### 3.5.3 Functional characterization of DEGs

**Figure 6:**
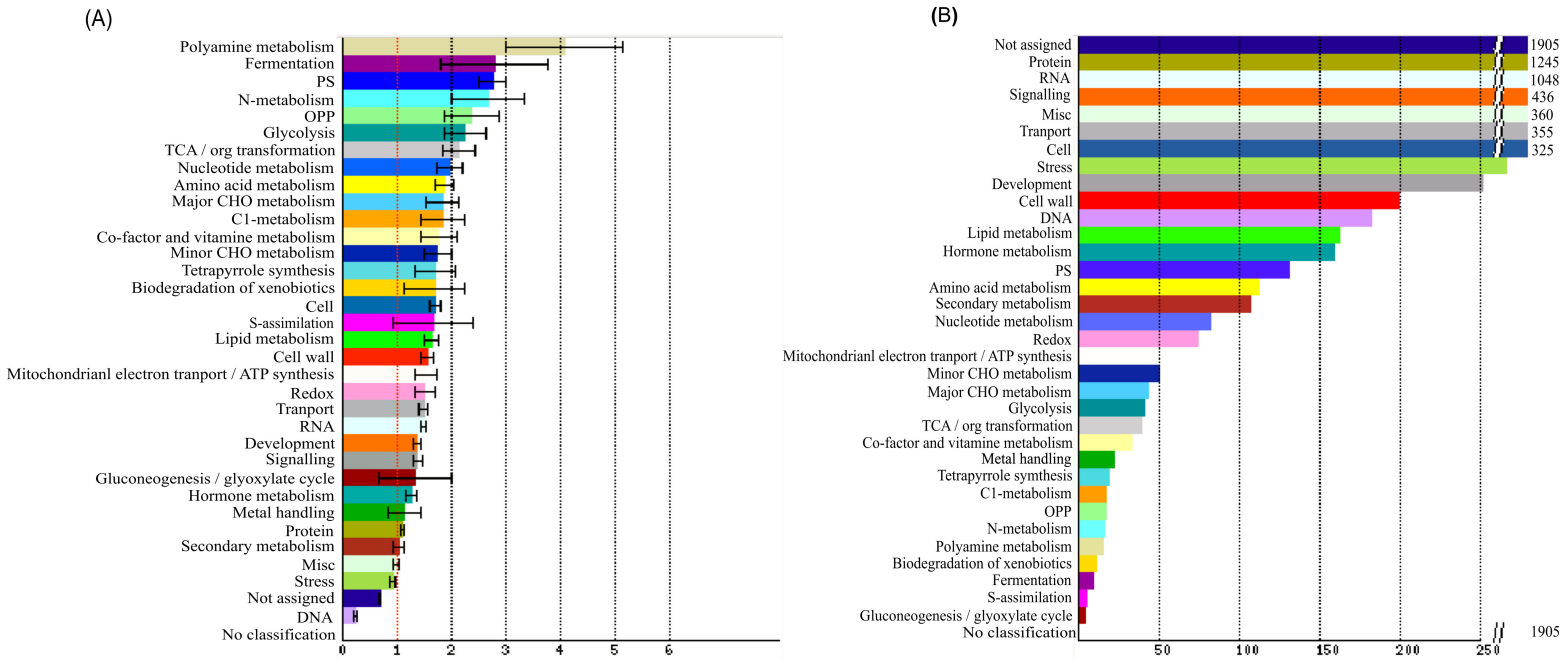
Functional classification of DEGs. The vertical axis shows the functional categories (significant enrichment in bold) and the horizontal axis shows the gene frequency **(A)** and absolute number **(B)** respectively.

To further reveal the function of these DEGs (**Supplementary Table S5**), their orthologues in *Arabidopsis* were classified using the SuperViewer, which showed the absolute number and normalized frequency for each class. These DEGs were classified into a total of 34 classes (**Figure 6**; **Supplementary Table S7**), of which 31 were enriched in significant level, suggesting that they might have diverse functions.

For normalized frequency, polyamine was the highest (4.09), followed by fermentation (2.80), photosynthesis (2.77), nitrogen metabolism (2.68), OPP (2.39), glycolysis (2.26) and TCA/ORG transformation (2.15), while other 26 classed showed a medium frequency between 0.7 and 1.99, and DNA was the lowest class (0.25).

For absolute number, protein (1245) and RNA (1048) were the most two classes, followed by signalling (436), miscellaneous (360), transport (355), cell (325), stress (266) and development (251), while tother 25 classes were relatively small in number (<200). The protein class comprised eight sub-classes, i.e. amino acid activation (38), synthesis (297), targeting (141), postranslational modification (240), degradation (462), folding (35), glycosylation (25), assembly and cofactor ligation (7). The second largest set of RNA classes included four subclasses i.e. processing (152), transcription (37), transcription factor (786) and binding (73). It should be noted that, transcription factor subclass occupied the highest proportion (75%) among the RNA classes, indicating the important role of TFs in controlling ovule number.

It should be also noted that 159 DEGs were classified into the metabolic and signalling pathways of several phytohormones, including auxin (43), brassinosteroid (30), ethylene (28), gibberellin (17), abscisic acid (17), jasmonate (16), cytokinin (4) and salicylic acid (4), suggesting the complexity of phytohormones interactions in regulating ovule number. More importantly, the expression difference of DEGs of AUX, BR, CK, and GA pathway between two pools were generally consistent with their content difference (**Table 4**).

To explore the relationship between these DEGs and ovule number, DEGs were compared to the reported genes regulating ONPO (Qadir et al., 2021). The results showed that 54 DEGs were homologous to 32 reported genes regulating ONPO, such as *AG*, *PIN1* and *SEP3* (**Supplementary Table S5**). In addition, among a total of 7689 DEGs, 3518 (45.8%) belonged to functional categories of genes regulating ONPO (Qadir et al., 2021), including protein (1245), RNA (1048), signalling (436), miscellaneous (360), development (251), hormone metabolism (159), and tetrapyrrole synthesis (19).

### 3.6 Identification of candidate genes by integrating of linkage mapping and transcriptomic analysis

In the reference genome of DarmorV4.1, a total of 289 genes were found to be the homologues of known ovule number genes, by using BLAST analysis (**Supplementary Table S8**). Of these, 15 were located within the genomic regions of identified ONPO QTLs except for *qONPO.A06-1* (**Table 5**), which should be the underling candidate genes. In addition, the number of DEGs in each QTL region varied from 11 to 73, with the sum of 327 (**TSupplementary Table S5**). More importantly, three DEGs in the QTL region (*BnaA01g23050D*, *BnaA06g30720D* and *BnaC05g21200D*) were also homologous to the known genes regulating ONPO, which should be considered as the most probable candidate genes prioritized for further functional validation. For example, *BnaA01g23050D* was located in the genomic region of *qONPO.A01-1*, its expression abundance in H pool was 3.76-fold that of L pool.

**Table 5:** The candidate and differentially expressed genes in genomic regions of detected ONPO QTL.

QTLs name	Genomic region	DEGs	Candidate gene	Distance to peak SNP	Homologue in *A. thaliana*	Gene Name	Annotation
*qONPO.A01-1*	*14.404-18.831*	*25*	*BnaA01g23050D*	1.381	*AT1G60780*	*HAP13*	µ1 adaptin component of heterotetrameric protein complex that regulates protein sorting at the trans-Golgi network/early endosome.
*qONPO.A03-1*	17.771-19.599	24	*BnaA03g37960D*	0.150	*AT5G35750*	*HK2*	Encodes histidine kinase 2: cytokinin-binding receptor that transduces cytokinin signals across the plasma membrane.
*qONPO.A06-1*	0.222-1.320	11	*/*	/	*/*	*/*	/
*qONPO.A06-2*	*20.902-22.536*	*19*	*BnaA06g30720D*	0.386	*AT5G38970*	*CYP85A1*	Encodes a polypeptide involved in the C-6 oxidation of brassinosteroids.
			*BnaA06g34500D*	1.663	*AT2G01830*	*HK4*	Encodes histidine kinase 4: cytokinin-binding receptor that transduces cytokinin signals across the plasma membrane.
*qONPO.A07-1*	*10.069-13.816*	*42*	BnaA07g11660D	1.364	*AT1G19850*	ARF5	Encodes a transcription factor (IAA24) mediating embryo axis formation and vascular development.
*qONPO.A09-1*	*27.572-29.899*	*20*	*BnaA09g38650D*	0.351	*AT3G60660*	*ONA2*	Encodes spindle/kinetochore-associated-like protein
			*BnaA09g39350D*	0.023	*AT3G61630*	*CRF6*	Encodes one of the six cytokinin response factors, and belongs to the AP2/ERF superfamily of the transcriptional factors.
			*BnaA09g40400D*	0.505	*AT3G63440*	*CKX6*	Encodes a protein whose sequence is similar to cytokinin oxidase/dehydrogenase, which catalyzes the degradation of cytokinins.
			*BnaA09g40540D*	0.585	*AT2G26330*	*ER*	Homologous to receptor protein kinases. Involved in specification of organs originating from the shoot apical meristem.
*qONPO.A10-1*	*10.483-15.040*	*73*	*BnaA10g13520D*	2.375	*AT5G60690*	*REV*	*REVOLUTA* regulates meristem initiation at lateral positions. a member of a small homeodomain-leucine zipper family.
			*BnaA10g18480D*	0.138	*AT3G02310*	*SEP1*	MADS-box protein, binds K domain of AG *in vivo*.
*qONPO.C02-1*	*0.17-6.57*	*42*	*BnaC02g01710D*	1.609	*AT5G07200*	*GA20ox3*	Encodes a gibberellin 20-oxidase.
*qONPO.C05-1*	*9.976-16.869*	*38*	*BnaC05g18750D*	2.454	*AT1G23420*	*INO*	Member of YABBY protein family of putative transcription factors and required for polarity determination in the central part of the ovule.
			*BnaC05g21200D*	4.753	*AT1G24260*	*SEP3*	Encodes a member and forms heterotetrameric complexes with other MADS box family members and binds to the CArG box motif.
*qONPO.C05-2*	*40.489-42.268*	*33*	*BnaC05g46680D*	0.930	*AT3G05120*	*GID1A*	Encodes a gibberellin receptor.

## 4 Discussion

### 4.1 QTLs for ovule number per ovary in oilseed rape

Previously, about 30 QTLs of ONPO (**Supplementary Table S3**) have been reported by a few linkage or association mapping studies using SNP array in oilseed rape (Ahmad et al., 2023; Khan et al., 2019; Qadir et al., 2022). These ONPO QTLs were distributed in 14 of all the 19 linkage groups, which accounted for 1.22-17.38% of the phenotypic variance with additive effect ranging from -1.03 to 2.70. In this study, a total of 10 QTLs were identified in a DH population of 201 lines through linkage mapping across four environments, which were distributed on eight chromosomes, accounting for 7.0-15.9% of phenotypic variance (**Table 3**). Of these, two QTLs in A07 and C02 linkage groups (*qONPO.A07-1* and *qONPO.C02-1*) were repeatedly identified in different environments and showed a relatively large effect, which should be considered as major QTLs and important target for practical application and further gene cloning. The relatively low reproducibility of detected QTL was highly accordant with the relatively low heritability of ONPO due to larger environmental difference in comparison with previous studies (Ahmad et al., 2023; Khan et al., 2019). Furthermore, the comparative QTL analysis (**Supplementary Table S3**) indicated that four QTLs in the present study were overlapped with those reported previously (Khan et al., 2019; Yuan and Kessler, 2019; Ahmad et al., 2023), which should be important targets for maker-assisted selection. More importantly, the remaining six QTLs should represent novel loci for ONPO. Except for *qONPO.A07-1*, the additive-effect direction of other five loci were all positive, indicating that favorable alleles were mostly from high-ONPO parent 3S1235. In addition, the high-ONPO genotype of peak SNP markers of these loci can be used for the molecular improvement of over number.

### 4.2 Phytohormones are major players in regulating ovule number

The ONPO is affected by both genetic environmental factors, including growth conditions, flower position (Qadir et al., 2022) and size (Wetzstein et al., 2013), and nutrient availability (Chalcoff and Aizen, 2016). Our recent review article summarized the reported ovule number genes and its regulatory pathways, which demonstrated that four types of phytohormones (AUX, CKs, GAs and BRs) played a central role (Qadir et al., 2021). It involves many biological process including phytohormones synthesis, degradation, transport, perception, signal transduction and downstream response etc., which regulate ovule development and finally affect its number. In our recent study (Qadir et al., 2022), the abundances of ABA, BA, GA4, IAA and JA showed significant difference between the two pools of high and low ONPO lines from an association population. To further reveal the roles of different sub-types of phytohormones in regulating ovule number, 48 sub-types of the above-mentioned four types of phytohormones were measured in the current study, of which nine had significant differences between two pools of high and low ONPO lines (**Table 4**). In addition, a total of 159 DEGs between these two pools were classified into the functional category of phytohormone metabolism (**Supplementary Table S5**), which involved eight types of phytohormones (ABA, AUX, BR, CK, ETH, GA, JA and SA). More importantly, of the total of 54 DEGs homologous to known genes regulating ovule number, at least 22 (40.7%) were relevant to phytohormones, of which 14 were classified into hormone metabolism and 8 belonged to transcription factors that contained AUX and ETH response element. These research results highly indicated that phytohormones should play a major role in regulating ONPO.

### 4.3 Tight association between DEGs and ovule number

In the reference genome *Darmor-bzh*, 289 annotated genes (**Supplementary Table S8**) were the homologues of 68 reported genes regulating ONPO (Qadir et al., 2021). Of these 289 homologues, 54 (18.69%) were differentially expressed between the two pools of high and low ONPO lines (**Supplementary Table S5**). For an example, *BnaA01g23050D* is an orthologue of *HAP13*, and its down-regulation decreases ONPO (Wang et al., 2016). Among a total of 111479 annotated genes in the Darmor-bzh reference genome, only 7689 (6.86%) were differentially expressed between the two pools mentioned above (**Figure 4C**). The former proportion was nearly three folds of the latter, which suggested that these DEGs were more preferentially to ovule number genes. In addition, the reported ONPO genes were classified into eight functional categories (Qadir et al., 2022), including RNA (23), hormone metabolism (22), development (12), signalling (6), protein (4), miscellaneous (3), micro RNA (1) and tetrapyrrole synthesis (1). It should be noted that nearly half of these DEGs belonged to the abovementioned categories (**Supplementary Table S5**), which suggested that they are highly involved in known pathways regulating ovule number. These results strongly supported that the DEGs identified in current study were tightly associated with ovule number.

### 4.4 Candidate genes for ovule number QTL

Genetic variations are valuable resources for understanding the biological basis of important traits and developing novel varieties with favorable traits. In the current study, a total of 10 ONPO QTLs have been identified using a DH population that was genotyped by 50K SNP array and phenotyped in four environments. Through BLAST analysis with the reported genes regulating ONPO (Qadir et al., 2021), a total of 13 homologues were found in these QTL regions (**Table 5**), which should be considered as the direct candidate genes for ONPO QTL after the confirmation of genetic sequence differences between two parents. It should be noted that eight of the 13 homologues were relevant to phytohormones, of which six were key enzymes in the metabolism of phytohormones and two were auxin or cytokinin response factors. This result was highly accordant with the observed phytohormone content difference between the two pools of high and low ONPP lines (**Table 4**), which highlight the key roles of phytohormones in regulating ONPO in oilseed rape. In addition, a lot of studies have proved that the combination of QTL mapping and transcriptomic analysis is an efficient approach for screening candidate genes (Hussain et al., 2021). In the current research, a total of 327 DEGs were also located in the physical regions of detected ONPO QTLs, which should also be potential candidate genes for ONPO QTL (**Table 5**; **Supplementary Table S5**). More importantly, a few of genes were common between DEGs and ovule number homologous genes, which should be considered as the most important candidate genes. The subsequent research should focus on the fine-mapping of major QTL followed by functional verification of the candidate genes.

## 5 Conclusion

This study performed a systematically genetic, physiological, and molecular dissection of ONPO in oilseed rape. The study identified ten significant QTLs for ONPO, of which four identical and two major QTLs were repeatedly detected across multiple populations and environments, providing key targets for marker-assisted selection. In fact, the peak SNP markers for the two major QTL have been applied for patent and will be utilized in breeding program. The study revealed essential role of phytohormones in regulating ovule number, which was evidenced by significant differences of nine sub-type’s content in ovaries at ovule initiation stage between high and low ONPO lines and the preference of DEGs to known genes and pathways regulating ONPO. Through linkage mapping and transcriptomic analysis, the study identified 15 candidate genes underlying the ONPO QTLs, of which three DEGs should be the most likely ones and will be validated in upcoming research. These findings proposed valuable insights into the genetic basis and molecular mechanisms of ONPO, facilitating future gene cloning and genetic improvement. The identified QTLs and candidate genes offered promising targets for marker-assisted selection and gene editing, accelerating the development of high-yield oilseed rape cultivars to address worldwide demands.

## Data Availability

The datasets presented in this study can be found in online repositories. The names of the repository/repositories. and accession number(s) can be found below: https://www.ncbi.nlm.nih.gov/ , PRJNA1150986.
