# Supplementary figures and images for: Dissection of the genetic basis and molecular mechanism of ovule number per ovary in oilseed rape (Brassica napus L.)
Source: Front Plant Sci. 2025 Jan 28;15:1489490. doi: 10.3389/fpls.2024.1489490 (PMC11811079; doi:10.3389/fpls.2024.1489490)

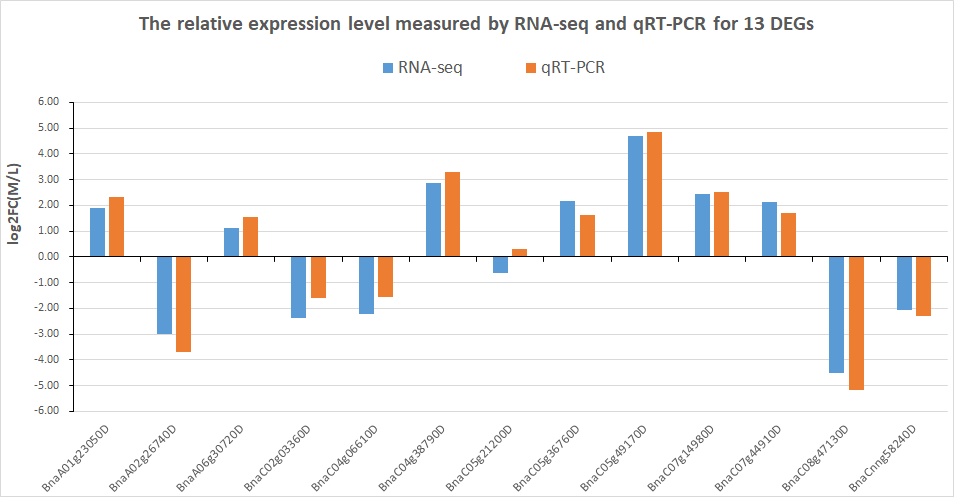

Supplement: Supplementary file 9 [file Image1.tif]
